# Monitoring and Evaluating Progress towards Universal Health Coverage in Chile

**DOI:** 10.1371/journal.pmed.1001676

**Published:** 2014-09-22

**Authors:** Ximena Aguilera, Carla Castillo-Laborde, Manuel Nájera-De Ferrari, Iris Delgado, Ciro Ibañez

**Affiliations:** 1 Universidad del Desarrollo Chile, Centro de Epidemiología y Políticas de Salud; 2 Ministerio de Desarrollo Social Chile; 3 FLACSO-Chile. Health Inequalities, Work and Access to Social Security of Informal Workers Project

## Abstract

This paper by Ximena Paz Aguilera and colleagues is a country case study for the Universal Health Coverage Collection, organized by WHO. It illustrates progress towards UHC and its monitoring and evaluation in Chile.

*Please see later in the article for the Editors' Summary*

This paper is part of the PLOS Universal Health Coverage Collection. This is the summary of the Chile country case study. The full paper is available as Supporting Information file Text S1.

## Background

With the establishment of the social security system in 1924, Chile started the path towards UHC. A key milestone was the creation of the National Health System in 1952, which offered public subsidized coverage for the poor [1]. Currently, after the partial privatization of social security in 1981 the health system is mixed, both in insurance and in service provision [2], and health insurance reaches 98% of the population, with 77% of this coverage by the public health insurance [3]. Regardless of this encouraging figure and a relatively good health situation, a significant burden of out-of-pocket (OOP) payment exists and the access to care has been described as noticeably inequitable between the public and private sectors [4],[5],[6],[7].

Defining UHC as a situation where all people obtain the health services they need without risking financial hardship from unaffordable out- of-pocket payments [8], this article reviews the case of Chile, with the aim of identifying indicators to monitor and evaluate UHC.

## Universal Health Coverage: The Policy Context

Since 2013, Chile has been ranked among high-income economies; life expectancy is 79 years [9]; poverty has fallen [10]; and education attainment has increased [11]. Despite all these positive trends, inequality still remains as one of the main challenges faced by the country [12].

In an attempt to tackle health inequities and to increase financial protection, in 2005 the Chilean government implemented an innovative health reform, with the central focus of the recognition of the right to health. This focus lead to the establishment of “explicit guarantees” for 80 prioritized health problems. The legal binding guarantee gives equal rights to the beneficiaries of the public and private sectors in four key aspects: access to health care (eligibility), timeliness of care (maximum waiting times), financial protection (maximum co-payments and deductibles), and quality of care (accreditation of facilities and professional certification) [13].

## Monitoring and Evaluation of UHC

Following main components of the UHC definition, we estimated or compiled two sets of indicators reflecting coverage of health services and financial protection coverage, set in a larger health services results framework [14]. Two priority groups were included for intervention coverage: Millennium Development Goals (MDGs) diseases and non-communicable diseases (NCDs). OOP health expenditure, as percentage of total household expenditure/income and as percentage of households facing catastrophic health expenditure, was used to reflect financial protection (FP) coverage. Household income, education, gender, and residence were used for equity disaggregation, adding ascription to public/private health insurance.

## Progress towards UHC in Chile

Coverage of health insurance is high, but there are still some gaps in needed health services and the quality is not sufficient to have effective treatments.

**Figure 1:**
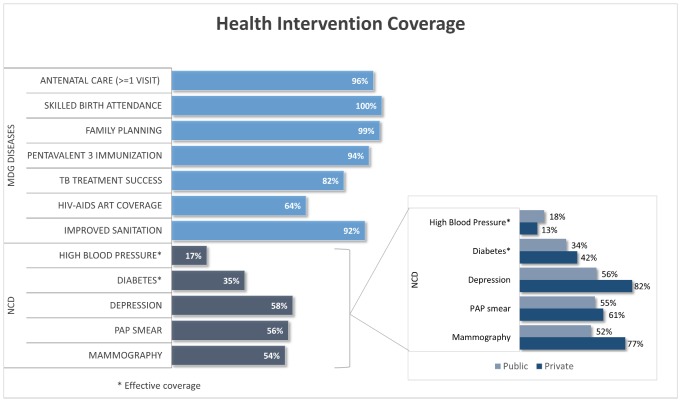
Coverage of interventions: tracer indicators for MDGs diseases and NCDs. Source: Based on Ministry of Health, National Health Survey 2009-10[15], and CASEN Survey 2011 [16].

NCDs are the leading causes of burden of diseases; however, their coverage is lower compared to MDGs diseases (infectious diseases, and maternal and child care, such as in antenatal care, family planning and skilled birth attendance), especially when measuring effective coverage (Figure 1). Additionally, prevalence of risk factors denotes minimum impact of preventive interventions. Equity disaggregation tends to show lower coverage for males, low-income quintiles, less-educated people, residents in rural areas, and people with ascription to public health insurance. Finally, some increasingly important diseases, such as dementias and others with catastrophic cost (like inherited metabolic disorders), are not adequately covered.

**Figure 2:**
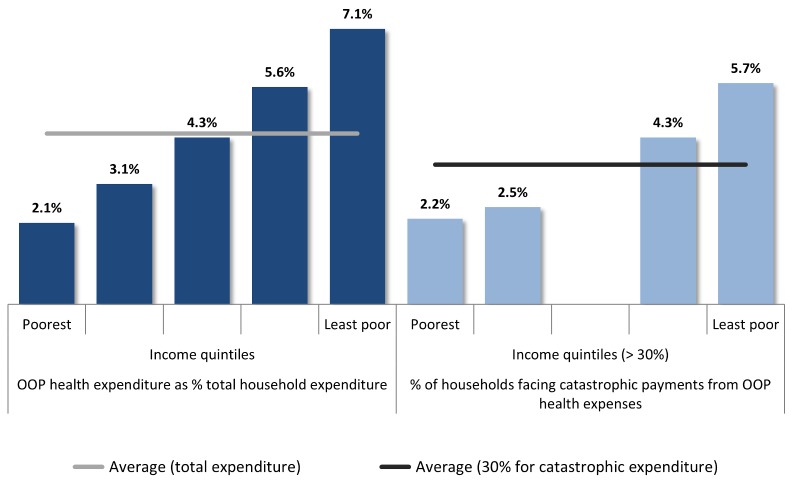
Financial protection tracer indicators. Source: Based on [17].

About 5% of the total expenditure at household level is devoted to paying for healthcare services and 1.9% of households face catastrophic health expenditures, with a 40% threshold (3.6% using 30% threshold), although both are progressive (Figure 2). Higher catastrophic costs among richer families could be related to the exemption of co-payment for the poorest quintiles, but also to equity gaps in complex services utilization.

## Conclusions and Recommendations

Over the last 90 years, the Chilean health system has moved towards UHC; however, structural constraints prevent further advancement and create gaps and inequities in terms of services coverage, which impact on health results [18],[19]. Moreover, it is observed that OOP payments have increased and the population still is not totally protected from the risk of facing catastrophic health expenditures.

Public spending is one of the lowest among OECD countries and OOP payments are the highest [20]. System fragmentation, at health insurance and provision levels, results in two realities: an underfunded and overwhelmed public sector and an elitist and increasingly expensive private sector.

UHC monitoring through indicators is feasible in Chile but requires a more robust information system. The Chilean health information system provides data for the required aspects. However, there are missing data for some priority health interventions and for equity disaggregation; at the same time, the consistency and periodicity of health surveys is not guaranteed. Additionally, FP figures only represent the city of Santiago. Overall, Chile has relevant and useful information that allows measuring and monitoring the path towards UHC.

Increasing resources available for healthcare, establishing solidarity among public and private sectors, and improving quality of care to expand effective coverage are key components towards achieving UHC in Chile.

## Supporting Information

Text S1 The full country case study for Chile.
(DOCX)

## Abbreviations

FP: financial protection
MDGs: Millennium Development Goals
NCDs: non-communicable diseases
OECD: Organization for Economic Co-operation and Development
OOP: out-of-pocket
UHC: Universal health coverage
