# Supplementary material for: Monitoring and Evaluating Progress towards Universal Health Coverage in Chile
Source: PLoS Med. 2014 Sep 22;11(9):e1001676. doi: 10.1371/journal.pmed.1001676 (PMC4171380; doi:10.1371/journal.pmed.1001676)
Supplement: Text S1 — The full country case study for Chile. (DOCX) [file pmed.1001676.s001.docx]

**Full Case Study: Monitoring and evaluating progress towards Universal Health Coverage in Chile**

Ximena Aguilera^1^, Carla Castillo-Laborde^2^, Manuel Nájera-De Ferrari^1^, Iris Delgado^1^, Ciro Ibañez^3^

^1^Universidad del Desarrollo Chile, Centro de Epidemiología y Políticas de Salud

^2^Ministerio de Desarrollo Social Chile

^3^Proyect Health Inequalities, Work and Access to Social Security of Informal Workers. FLACSO-Chile

*Corresponding author: Ximena Aguilera

Email: xaguilera@udd.cl

**This paper is the full country case study to accompany the summary paper “Monitoring and evaluating progress towards Universal Health Coverage in Chile” that is part of the Universal Health Coverage Collection. Not commissioned; externally reviewed.**

**Abstract:** In a context where Universal health Coverage is one of the possible development goals of the post 2015 agenda, comparable data at country level is required. The present article reviews the case of Chile, with the objective to identify UHC indicators. Two sets of indicators reflecting coverage of health services and financial protection were estimated, including equity disaggregation. Interventions for MDGs diseases and NCDs were used for health services coverage, while out of pocket expenditure and catastrophic health expenditure were used to reflect financial protection coverage. Health insurance coverage is 98%. Coverage for MDGs diseases is significantly higher compared to NCDs, especially when measuring effective coverage for high blood pressure and diabetes. Equity disaggregation show lower coverage for males, low-income quintiles, less educated, residents in rural areas and people with ascription to public health insurance, with the exception of HBP, where public overcomes private insurers. 5% of the total expenditure/income at household level is devoted to pay for healthcare services and 1.9% of households face catastrophic health expenditures (threshold: 40% household capacity to pay), although both are progressive. Health system has moved towards UHC, but there are structural constraints that create gaps and inequities both in availability of resources and in utilization of health care. Public expenditure on health is one of the lowest among OECD countries and OOP is the highest. Fragmentation at pooling and provider levels results in two realities: an underfunded and overwhelmed public sector and an elitist and increasingly expensive private sector. To increase the resources available for health, to establish solidarity among public and private sector and to improve quality of care to expand effective coverage, seems key components towards UHC in Chile. UHC monitoring through indicators is feasible but requires robust information systems, periodic health surveys and a focus in vulnerable population, especially in rural areas.

**Summary Points:**

1. Over the last 90 years the Chilean health system has moved towards UHC; however, structural constraints prevent further advance and create gaps and inequities in terms of services coverage, impacting health results.
2. NCDs are the leading causes of burden of diseases; however their coverage is lower compared to MDGs diseases, especially when measuring effective coverage, reflecting quality of care issues.
3. Moreover the Chilean population is not adequately protected from the risk of facing catastrophic health expenditures.
4. System fragmentation, at pooling and provider levels, results in two realities: an underfunded and overwhelmed public sector and an elitist and increasingly expensive private sector.
5. UHC monitoring through indicators is feasible but requires a more robust information system. The Chilean health information system provides data for all required aspects. However there are missing data for some priority health interventions and for equity disaggregation; at the same time the consistency and periodicity of health surveys is not guaranteed.
6. Increasing resources available for health, establishing solidarity among public and private sectors and improving quality of care to expand effective coverage, are key components towards UHC in Chile.

**1. Background**

To improve the health of the population is the primary goal of any health system, but it is not the only one. Fairness in financial contributions and responsiveness to people’s expectations have also been recognized as important goals of a health system[1]. In this context, the World Health Assembly Resolution 58.33 urged the countries to develop their health systems to ensure all people have access to necessary services while providing protection against financial risk[2], this has been defined as Universal Health Coverage (UHC). In 2012, the UN called upon the countries to scale up efforts to accelerate the transition towards this goal[3].

Considering that UHC has been listed as one of the possible development goals of the post 2015 agenda[4], it is necessary to operationalize the concept, and afterwards counting on comparable data at the country level. In this context, the particularities of the Chilean health system, present an interesting case to study the dimensions of UHC.

Since 1924 Chile has made systematic efforts in order to achieve UHC. A major milestone was the creation of the national health system in 1952[5], which offered public subsidized coverage for the poor and a countrywide network of public primary health care centers (PHC) and hospitals. After deep reforms including partial privatization of social security, current health system is considered mixed, both in insurance as in service provision[6]. Currently 98% of the population has health insurance coverage, 77% by the public health insurance[7]. Despite this high coverage and a reasonably good health situation, there is a significant burden of out-of-pocket payment and the access to care can be described as noticeably inequitable among public and private sector[8, 9, 10, 11].

The present article reviews the case of Chile; the objective is to identify a set of indicators in order to characterize the situation regarding UHC. The document contain five sections: 2) UHC: the policy context; 3) Monitoring and evaluation for UHC; 4) Progress towards UHC in Chile and 5) Conclusions and recommendation.

**2. Universal health coverage: the policy context**

Chile has a population of 17 million, highly concentrated in urban areas (87%) with a life expectancy of 79 years in 2011[12, 13]. In 2010 Chile joined the OECD and from 2013 it is ranked as a high income economy by the World Bank (GNI per capita of PPP $ 21,590 in 2012). The economic growth observed during the period 1960-2012 (GDP grew from $550 to 15,000 PPP)13, as well as targeted social policies and programmes implemented through the last decades, have had important consequences in the development indicators. In fact, the population below poverty and extreme poverty line has fallen from 38.5% and 13% in 1990 to 14.4% and 2.8% in 2011 respectively[14]. In parallel, the average years of schooling increased from 9 to 10.6 years between 1990 and 2011 (8 years in rural and 10.9 years in urban areas for 2011). Primary and secondary education is universal, while technical and university enrolment augmented, persisting differences between urban and rural areas[15].

Despite these advances, inequality still remains as one of the main challenges faced by the country. The GINI coefficient increased considerably between middle 50’s and 80’s, reaching its highest level (0.6) in 1987[16] and falling to a level that has been maintained during the last decades (0.55 approx.)[17]. Inequalities are also present in health in utilization and health impact indicators[8, 9, 10, 11, 18, 19].

Throughout its history Chile has made systematic efforts in order to achieve UHC[5, 18, 19, 20, 21]. As the result of the reforms performed, the Chilean health system can be described as a mixed system, with public and private insurance performing the collection, pooling and purchasing functions; and public and private providers responsible for the provision of health services[6]. The coexistence of both sectors is consequence of the 1980 social security reform, which allowed the introduction of private competing health insurance funds (ISAPREs) as an alternative to the national social insurance fund (FONASA). The historical perspective is reviewed in Box S1.

| **Box S1: Historical perspective of the Chilean Health System**  16th and the 19th centuries: emergence of several non –for- profit health organizations (public and private).  1886: Enactment of the Statute of Charity Board (“Junta de Beneficencia”) in order to unify local health care organizations and hospitals.  Early 20th century: increment of the direct state investment dedicated to building hospitals in different cities of the country (leaving less space to charity in health financing).  1918: Approval of the first Sanitary Code of the Chilean Republic, establishing the Directorate of Health, in charge of public health functions, such as vaccination, public disinfection, among others.  October 1924: Creation of the Ministry of Hygiene, Assistance, Labor and Social Welfare, which over the years became multiple ministries, one of them the Ministry of Health.  1924: Creation of the Worker’s Compulsory Insurance (“Seguro Obrero”), to cover the risks related to illness, disability, aging and death.  1925: The Political Constitution recognizes the "the right to health”.  1938: Approval of the laws: N°6.174 of Preventive Medicine (i.e. systematic examinations to workers) and N°6.236 of Mother and Child (i.e. protect mothers and children from delivery difficulties).  1942: Establishment of the SERMENA (National Medical Service for Civil Servants for its acronym in spanish), in order to provide coverage to the civil servants.  1952: Creation of the National Health Service (SNS for its acronym in spanish), merging the various existing bodies related to public health. The SNS became the cornerstone of Chilean health system, allowing significant progress in the planning and administration of the healthcare, progressively increasing health coverage, focused primarily on maternal and child health and communicable diseases.  The 70’s: The emphasis was placed on maternal and infant health, with a significant impact in reducing mortality, perinatal and child mortality in the country.  1979: The Ministry of Health System was restructured (merging the SNS and the SERMENA). Emerging the new National System of Health Services, that unified the Health Services – SNSS (i.e. in charge of the provision health care), the FONASA or National Health Fund (responsible for the financing function), the CENABAST or National Supply Center and the National Institute of Public Health.  1980: Beginning of the municipalization of primary health care centers (giving its administration to local governments), which facilitated inequity in availability of resources among rich and poor local governments.  1980: The Political Constitutions states the “right to protection of health”, and recognizes the role of the private insurance and providers.  1981: Creation of the ISAPREs or Health Insurance Institutions.  2000s: Health Reform, including the establishment of the Explicit Health Guarantees (GES) in 2005.  2000: Establishment of the National Health Objectives for the decade 2000-2010.  2006: The Program “Chile Grows Along With You” (Chile Crece Contigo) was launched. A comprehensive child protection system, created to offer differential support for children between 0 and 4 years old and also to their families.  2011: Establishment of the National Health Strategy for the decade 2011- 2020. Defined the four National Health Objectives: improving population’s health; decreasing health inequalities; increasing population’s satisfaction; and assuring the quality of health services. The strategy includes 9 Strategic Objectives (with goals, expected results and strategies indicators) related to communicable diseases, NCDs, risk factors, life cycle, social determinants and equity, environment / food and work, strengthen the system, access and quality of healthcare, and emergency and disasters.  2011: The Program “Choose to live healthily” (Elige Vivir Sano) was released. Inter-ministerial program resulting from a public-private Alliance, its objective is to persuade the Chilean people to change their habits, based on social marketing to intervene on four aspects: healthy diet, physical activity, spending more time outdoors and more time with the family. |
| --- |

In an attempt to reduce inequities among public and private health sectors, in 2005 the Chilean government implemented an innovative health reform, whose central focus were the recognition of the right to health. This lead to the establishment of “explicit health guarantees” for 80 prioritized health problems, which represents the main causes of burden of diseases and injuries in Chile. The legal binding guarantee gives equal rights to the beneficiaries of both sectors across the whole Chilean territory ^[22]^. Box S2.

| **Box S2: The 2000’s Health Reform: Explicit health guarantees AUGE**  During the last decade Chile faced a major health reform whose central focus were the recognition of the right to health, equity, solidarity, efficiency, and social participation in health.  The reform was supported by five laws (i.e. N° 19,888; N° 19,895; N° 19,937; N° 19,966; N° 20,015), one of them regarding the funding of the reform (i.e. an increase in VAT) and two of them regarding the regulation of the ISAPREs. The fourth law established a set of regulations related to the governing faculties of the Ministry of Health and the regulation and enforcement powers granted to other institutions (e.g. the Superintendence of Health). Finally, the law N° 19.966 established the ‘Explicit Health Guarantees’ (i.e. GES, for its acronym in Spanish), which are considered the corner stone of the reform[22].  The GES set explicit guarantees associated to the diagnosis, treatment and follow up of 80 prioritized health problems, which represent the main causes of burden of diseases and injuries (e.g. hypertension, diabetes, depression, acute myocardial infarction, stroke, traumatic brain injury, schizophrenia, Parkinson’s disease, alcohol and drug addiction, multiple sclerosis, and breast cancer). The guarantees cover four areas: access (i.e. who is eligible?), timely access (i.e. for eligible beneficiaries the law establishes maximum waiting times), financial protection (i.e. it establishes maximum copayments and deductibles) and quality (i.e. accreditation of facilities and professional certification).  In addition, through the same legal mandate, the MOH defines yearly coverage goals to all health insurers for preventive screenings throughout the life cycle (EMPA). Coverage Goals range from 15% coverage for lipid profile (people >40 yo), up to 100% for newborn screening (hip dysplasia, phenylketonuria and congenital hypothyroidism).  The fact that the guarantees are the same for beneficiaries from FONASA and ISAPREs is considered one of the main achievements of the reform. Another important achievement, in a country where there was not a general drugs reimbursement policy, is the inclusion of the medicines associated to the treatments of the prioritized health problems. The latter means that, for instance, highly prevalent, chronic and with intensive drugs treatment problems, such as hypertension and diabetes people who choose to get attention through the GES will have financial coverage also for medicines (people in FONASA or ISAPRE may opt to get attention out of the guarantees through the “free choice” modality).  The number of health problems covered by the GES has been growing gradually; starting with 25 in 2005, 40 in 2006, 56 in 2007, 69 in 2010, and reaching 80 in 2013 (see the complete list in Box S5, end of document). |
| --- |

Health insurance contribution is compulsory for formal workers, but they may ‘opt out’ of the FONASA, choosing to enroll in the ISAPREs. The ISAPREs are financed by individual risk-related contributions, with a minimum of 7% of the salary; however, the average contribution is 10% depending on the health plan contracted, and there are more than 12,000 different insurance plans [23].

NB: An insurance plan defines the financial coverage for a package of health services, depending on whether the services are outpatient or inpatient and the type of provider (i.e. free choice or preferred provider). As a minimum any ISAPRE plan must offer the same coverage offered by FONASA in its free choice modality – MLE.

On the other hand, FONASA is financed by a combination of the 7% salary-based contribution of formal workers and general taxes, also covering the worse off not contributing to the system.

In 2012 FONASA covered 77% of the Chilean population and 13 ISAPREs covered 18%[7]. The remaining 5% correspond to uninsured population (2%) and population covered by the Armed Forces health insurance (3%). In parallel, the Occupational Health Insurance covers the formal workers against occupational diseases and work accidents. The funding to this system is based on the employees’ mandatory contributions, collected by the Mutual Security institutions, providing health care through their own facilities. In 2009, approximately 2.5 million workers were covered against such events[24].

On the provider’s side, the National System of Health Services (SNSS) coordinates the public sector facilities; composed by the 29 autonomous Health Services and the municipal system of primary health care (PHC). FONASA finances two modalities of provision, the institutional modality (MAI for its acronym in Spanish) and the free choice modality (MLE). The former considers the provision by the public facilities; while MLE considers the provision by private facilities that have previous contracts with FONASA. In the public sector, the provision is organized in health care networks with levels of complexity, beginning with PHC facilities spread throughout the national territory. In the private sector, the provision is delivered by private facilities either trough free choice or preferred providers.

**3. Monitoring and evaluation for UHC**

In the context of the National Health Objectives 2000-2010, in preparatory phases of the 2005 health reform, the MOH implemented a plan to improve health information system, in order to support the definition of health priorities and also monitoring the future impact of health guarantees. This lead to the implementation of a system of routine health surveys: the Quality of Life and Health Survey (2000-2006); the National Health Survey, with biomarkers (2003-2009/2010); the Satisfaction and Out-of-Pocket Payment Survey (2006) and the reformulation of the health module of the National Socioeconomic Survey (2000-2003-2006-2009-2011). In parallel, both public and private health insurers developed information systems to capture the utilization statistics for guaranteed health services and monitor the guarantee comply.

The existence of a new National Health Strategy 2011-2020 that defines goals for broad health objectives (improve health population, decrease health inequalities, increase population’s satisfaction, and assuring the quality of health services) and their strategic objectives also imposes the challenge of monitoring the performance of the health system.

All these previous efforts have facilitated the measurement of key aspect considered in the operational definition of UHC (Box S3). To this purpose, in a first stage, considering the WHO conceptual framework of UHC, the present review attempts to characterize the Chilean health system trough the three basic dimensions: who is covered, services covered and proportion of cost covered[32].

| **Box S3: Sources of information**  **Health Sector:**  Ministry of Health (MoH): The MoH is in charge of producing the official statistics of the sector, providing information on births, mortality, hospital discharges, outpatients consultations, emergency care, immunizations, resources for health (health workers and facilities), among many others. The feasible breakdowns for these data depend on the way the information is originally registered. At the epidemiological level, the MoH also provides information of health results, based on the routine surveillance of communicable and non-communicable diseases, and the development of health population surveys. Also, the specific diseases National Programmes, such as the HIV/AIDS and Tuberculosis (TB), manage their own monitoring systems. Finally, the MoH produces economic statistics for the public health sector (at the funding and provider levels), and for the private sector (at the funding level). Some of the relevant population surveys are:  -Quality of Life and Health Survey (ENCAVI 2000 - 2006): household survey applied in order to study the Chileans’ perception about their quality of life and health, as well as their determinants (e.g. life styles, income, education, disabilities). The results are representative at the national, regional and urban/rural levels (>15 years of age; in 2006 included a module for infants under 15 years of age)[25, 26].  -National Health Survey (ENS 2003 – 2009/10): household survey that includes biomedical measurement of relevant health conditions (e.g. diabetes, hypertension, HIV/AIDS, risk factors), in individuals over 15 years of age. The results are representative at the national, regional and urban/rural levels; and allowing several breakdowns for the data (e.g. regional, urban/rural, educational level, income quintiles, public/private insurance, age, ethnicity, gender)[27, 28].  -Satisfaction and Out-of-Pocket Payment Survey (2006): household survey developed, in the context of the WHO World Health Survey, in order to measure the degree of satisfaction with the health system and the level of out-of-pocket payments for health services. The results are representative at the national and urban level (>18 years of age)[29, 30].  -National Survey of Employment, Labor, Health and Quality of Life of Workers in Chile (2009): the survey was applied on the occupied population (>15 years of age) and the unoccupied population with a job during the 12 months before the survey. The objective was to address the employment conditions of the Chilean workers. The results are representative at the national, regional and urban/rural level[24].  In addition to the MoH, other health sector institutions that produce relevant information are the Superintendence of Health (SOH) (at the insurance and providers level) and the FONASA (e.g. coverage, financing, expenditure)..  **Sources outside the health sector:**  -Socioeconomic Characterization Survey (CASEN, 1985-1987-1990-1992-1994-1996-1998-2000-2003-2006-2009-2011): household survey developed by the Ministry of Social Development in order to learn about the socioeconomic situation of the households, including a module on health that collects information regarding insurance affiliation and utilization of health services, allowing several breakdowns for the data (e.g. by public/private insurance, income, age, gender, ethnicity).  -National Youth Survey (1994-1997-2000-2003-2006-2010-2012): the survey is developed by the National Youth Institute, considering individuals between 15 and 29 years of age, in order to provide a diagnosis and analysis of the youth in the country. The survey includes a module on reproductive health and sexuality. The results are representative at the national, regional and urban/rural level[31].  -Household Budget Survey (EPF 1956/57–1968/69–1977/78–1987/88–1996/97–2006/07-2011/12): Household survey developed by the National Institute of Statistics (INE) in the context of the measurement of the Consumer Price Index. The EPF collects information on the household expenditure (including expenditure on health), allowing the estimation of the out-of-pocket expenditure as well as the incidence of catastrophic payments. In general, the results are representative at the Great Capital or Great Santiago level, although the two last surveys have considered all the regional capital cities. The analyses currently available consider the results for 2006/07, the results for 2012 have been recently released.  Other relevant secondary sources of information are the studies developed by the academy, consultants or other institutions, either funded by the National Fund for Research and Development (FONIS), commissioned by the MoH, or self funded. Finally, there is also official information available through international sources such as the World Bank Open Data, and the OECD Statistics (as Chile has recently become a member). |
| --- |

In a second stage, following the main components included in the UHC operational definition, two main sets of indicators reflecting coverage of health services and financial protection were estimated, set in a larger health services result framework considering inputs, outputs, outcomes and impact[33]. At this point, an advisory group was created in order to discuss the conceptual framework. Afterwards, feasible indicators (and their breakdowns) covering each dimension in the Chilean context were identified. Finally, the data was collected for each of the indicators, selecting, according to availability, the sources in the following order: official information reported by the Ministry of Health; official databases population surveys made by the government; official information reported by other related institutions and other sources (e.g. indexed articles or grey literature).

For intervention coverage two priority groups were included: MDGs, that include infectious diseases and Maternal, Neonatal and Child health, coinciding with Group I in the BoD; and NCDs or BoD Group II, including chronic conditions. There were several potential tracer indicators for interventions oriented to MDGs diseases that are cost-effective, such as immunization, where is easy to identify the denominator, or population needing the intervention. These indicators are routinely monitored, trough administrative registries. On the other hand, indicators for NCDs are less available; it requires health surveys to identify population in need and also to estimate effective coverage, in order to include a measure of quality. Coverage indicators for injuries were not possible to identify, only occupational health insurance coverage as a proxy.

For financial risk protection, three main indicators were estimated: at the macro level, the % of health expenditure finance through OOP expenditure; at the micro level, the % of the household total expenditure (or income) devoted to OOP health payments, and the % of households facing catastrophic payments from OOP expenses (>30% and <40% of household capacity to pay).

Recommended breakdowns were used for equity disaggregation: household income, gender, education and residence. Health insurance coverage (public/private), was added because its relevance to reflect social inequity in Chile.

**4. Progress towards UHC in Chile**

The three dimensions of UHC:

Who is covered? Approximately 98% of the Chilean population have health insurance coverage, considering both public and private insurers. Family income mainly decides the choice of insurer; however the population not covered is not only the poor. Instead, the population without coverage is about 10 percent of each income decile[34]. Occupational health insurance coverage is 41% of the formal and independent workers [24].

What Services and how much of the cost is covered? There is not a simple answer. Health services could be grouped into three different categories, and each of them have different proportion of the cost covered: 1) Public health goods, with universal coverage and 0 copayment, comprises all the public health services: health promotion, prevention programmes and the control of epidemic prone diseases. 2) Health problems with explicit legal guarantees (GES-AUGE): both public and private sector have maximum copayment and deductible, and there is a legal decree that defines the package of interventions associated to each of the 80 prioritized problems. 3) For the other health problems and conditions, copayments differ in both sectors. In the public sector copayments are determined by family income and type of provider (Table S1), while in the private sector copayments are defined on each health plan. Some services for increasingly important diseases, such as dementias and other with catastrophic cost, like inherited metabolic disorders, are not satisfactorily covered.

| FONASA group | Proportion of beneficiaries | Proportion of total population | Gross monthly income (US$) (2013) | Other characteristics | Co-payments | | | | | |
| --- | --- | --- | --- | --- | --- | --- | --- | --- | --- | --- |
|  |  |  |  |  | MAI: Institutional Providers (Public Health Sector) | | | | MLE: Private Providers (Free-choice) | |
|  |  |  |  |  | Primary Health Care | In-patients and specialist consultation | Oral health | 80 health problem under GES | Outpatient care | In-patient care |
| A | 24% | 19% | Indigents | Indigents / basic solidariity pensions beneficiaries (pensión básica solidaria) / family allowance beneficiaries (subsidio familiar) | 0% | 0% | 0% | 0% | not allowed | not allowed |
| B | 37% | 28% | < 432 |  | 0% | 0% | 30% | 0 - 20% with an annual deductible depending on socioeconomic level, number of health problems and affiliation | 50 - 60% | up to 75% |
| C | 17% | 13% | 432 -631 | If the contributor has more than 3 dependents beneficiaries pass to group B | 0% | 10% | 50% |  | 50 - 60% | up to 75% |
| D | 22% | 17% | > 631 | If the contributor has more than 3 dependents beneficiaries pass to group C | 0% | 20% | 80% |  | 50 - 60% | up to 75% |
| Total | 100% | 77% |  |  | 0% |  |  |  |  |  |

**Table S1**. FONASA groups and financial coverage

Data Source: authors based on FONASA (2013)

Note: Observed Exchange Rate (average Jan-Aug 2013) 1US$ =486,21 Chilean peso

UHC Monitoring Indicators: Interventions for MDGs Diseases

Seven intervention areas were selected, reflecting services that range from non/personal to secondary or tertiary care (Table S2, end of file), most of them are either public goods or part of established programmes in the PHC. Coverage fluctuates from 64% for HIV-AIDS ART to 100% for skilled birth attendance (Figure S1).


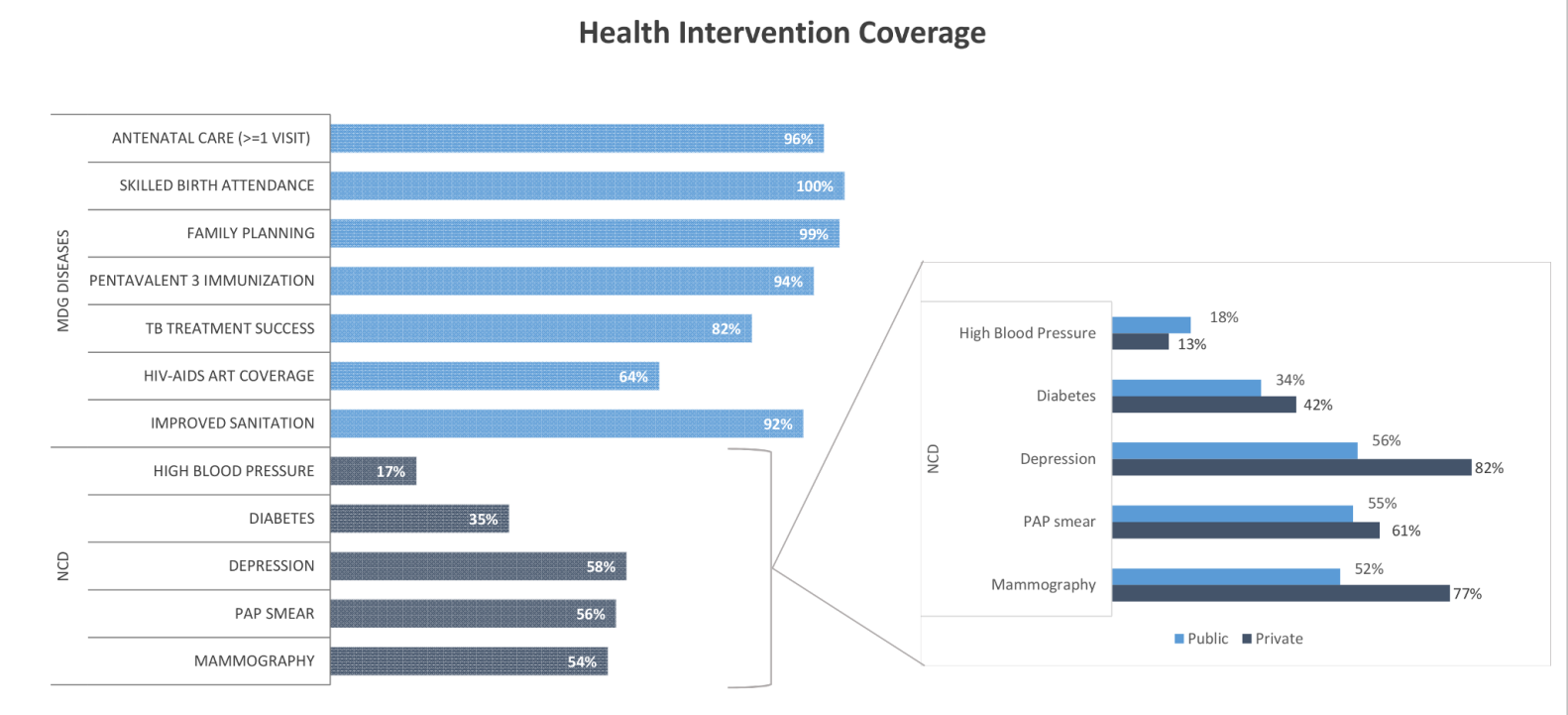


**Figure S1**: Coverage of Interventions: Tracer Indicators for MDGs diseases and NCDs

Data Source: Ministry of Health and CASEN Survey 2011.

Maternal and child care reach the highest coverage. Immunization is also high, but has been declining over the last years. TB treatment success differs according to administrative regions; the lowest treatment success is obtained in the region with the highest TB incidence, and the failures have been attributed to programme underfunding[16, 36]. HIV-AIDS ART treatment has financial protection, but the coverage is comparatively lower, mainly due to the application of an updated clinical guideline, which define a higher proportion of HIV patients needing treatment. Finally, despite the high access to water and sanitation, and the government subsidy for vulnerable population in urban and rural areas, sanitation coverage is significantly lower in rural houses. Equity gradients are also seen according to household income and household´s head education and gender. The amount of subsidies seems not enough to guarantee equitable coverage to these interventions.

UHC Monitoring Indicators: Interventions for NCDs

Five indicators were selected to characterize NCDs interventions, trying to reflect main causes of burden of diseases and cost-effective interventions, performed in different levels of care (Table S2, end of file, and Figure S1). Coverage for NCDs interventions is lower compared to MDGs diseases. The minimum is obtained when computing effective coverage (EC)[37], indicating the proportion of people with normal parameters (BP or glycaemia), among those in need of treatment for hypertension or diabetes. However effective coverage for both HBP and Diabetes has slightly improved since 2003.

For instance, less than one in five people reach normal blood pressure (BP) among those in need. The main equity gradient is related to gender; men have less than half EC compared to women. This is the only intervention where public sector coverage overcomes private sector, although EC is very low in both. EC for diabetes treatment is better, but around 40%. Main equity gaps for Diabetes are related to rural residence and gender.

Depression treatment has higher coverage compared to previous chronic conditions, but because effectiveness is not included. It only tells the proportion of people with depression that are under treatment. People in ISAPREs reach the highest coverage, while males in average have the lowest. Coverage for cervical cancer screening is under 60% and is lower in poorer, less educated women and in FONASA beneficiaries. Beginning in 2014 HPV vaccine will be incorporated as a public good to the expanded programme on immunization. On the other hand Mammography coverage seems too high considering its relative recent introduction. Equity gaps for mammography are related to health insurer, income and education.

As it was mentioned in Box S2, the explicit guarantees also include the obligation to perform screening examination trough the lifecycle (EMPA), with specific coverage goals to promote early diagnosis and to report them annually. The last report available only has ISAPREs screening coverage, showing results below the goals in the vast majority of age groups[38]. Data for FONASA was obtained from MOH for only two target groups, reaching goals in both (Table S3).

| Population group and specific screening intervention | Description | Coverage goals | EMPA Coverage | |
| --- | --- | --- | --- | --- |
|  |  |  | ISAPREs | FONASA |
| Newborns |  |  |  |  |
| Preventive Medicine examination | screening for phenylketonuria, congenital hypothyroidism and hip dysplasia. | 100% | 36.36% | N/A |
| Lactating |  |  |  |  |
| Hip dysplasia screening |  | 60% | 1.19% | N/A |
| Children under 4 years |  |  |  |  |
| Preventive Medicine examination | screening for amblyopia, strabismus, and defects in visual acuity; oral habits, and overweight and obesity | 60% | 0.67% | N/A |
| People Over 15 |  |  |  |  |
| Preventive Medicine examination | screening for drinking problems, hypertension, overweight and obesity, smoking, diabetes, syphilis and Tuberculosis. | 25% | 13.88% | N/A |
| Pregnant |  |  |  |  |
| Preventive Medicine examination | screening for drinking problems, hypertension, overweight and obesity, smoking, diabetes, syphilis, HIV and Urinary tract infections. | 100% | 15.38% | N/A |
| Women between 25 to 64 years |  |  |  |  |
| Preventive Medicine examination | screening for drinking problems, hypertension, overweight and obesity, smoking, diabetes, syphilis, tuberculosis, dyslipidemia and cervical cancer. | 75% | N/A | 39.40% |
| Cervical cancer screening |  | 75% | 68.98% | N/A |
| Women between 50 to 54 years |  |  |  |  |
| Brest Cancer screening |  | 25% | 84.64% | N/A |
| Men Between 25 to 64 years |  |  |  |  |
| Preventive Medicine examination | screening for drinking problems, hypertension, overweight and obesity, smoking, diabetes, syphilis, tuberculosis and dyslipidemia. | 25% | N/A | 14.92% |
| People over 40 years |  |  |  |  |
| Dyslipidemia |  | 15% | 77.32% | N/A |
| People over 65 years |  |  |  |  |
| Preventive Medicine examination | screening for drinking problems, hypertension, overweight and obesity, smoking, diabetes, syphilis, tuberculosis, dyslipidemia and functional autonomy. | 40% | N/A | 43.02% |
| Functional autonomy screening |  | 40% | 0.49% | 43.02% |
|  |  |  |  |  |
| N/A Not Available |  |  |  |  |

**Table S3**: Screening Coverage (EMPA) Chile 2011-2012

Data Source: Authors based on SoH and MOH –Chile.

UHC Monitoring Indicators: Financial Protection

OOP expenditure corresponded to 38.3% of the total health expenditure (THE) in 2012 that almost duplicate the average of OECD countries[39]. At the micro level, in 2007 the households devote about 5% of their total expenditure/income to pay directly for health services, differing importantly according to health insurer. In fact the average for ISAPRE is 6.1%, while for FONASA is 3.8%[40].

The high proportion of OOP is mainly due to: drugs spending; the use of MLE in the public health insurance and to additional contribution (over 7% of salary) to private insurers for more comprehensive health plans.

Although, the proportion of households facing catastrophic payments was 3.6%, when considering a threshold of 30% (1.9% when considering a 40% threshold), OOP expenditure is progressive, showing higher payments for the better-off income quintiles[41]. So the risk of impoverishment due to health is considerably higher for this group (Figure S2). The same study showed that OOP increased significantly in 10 years, while catastrophic spending decreased slightly[41].


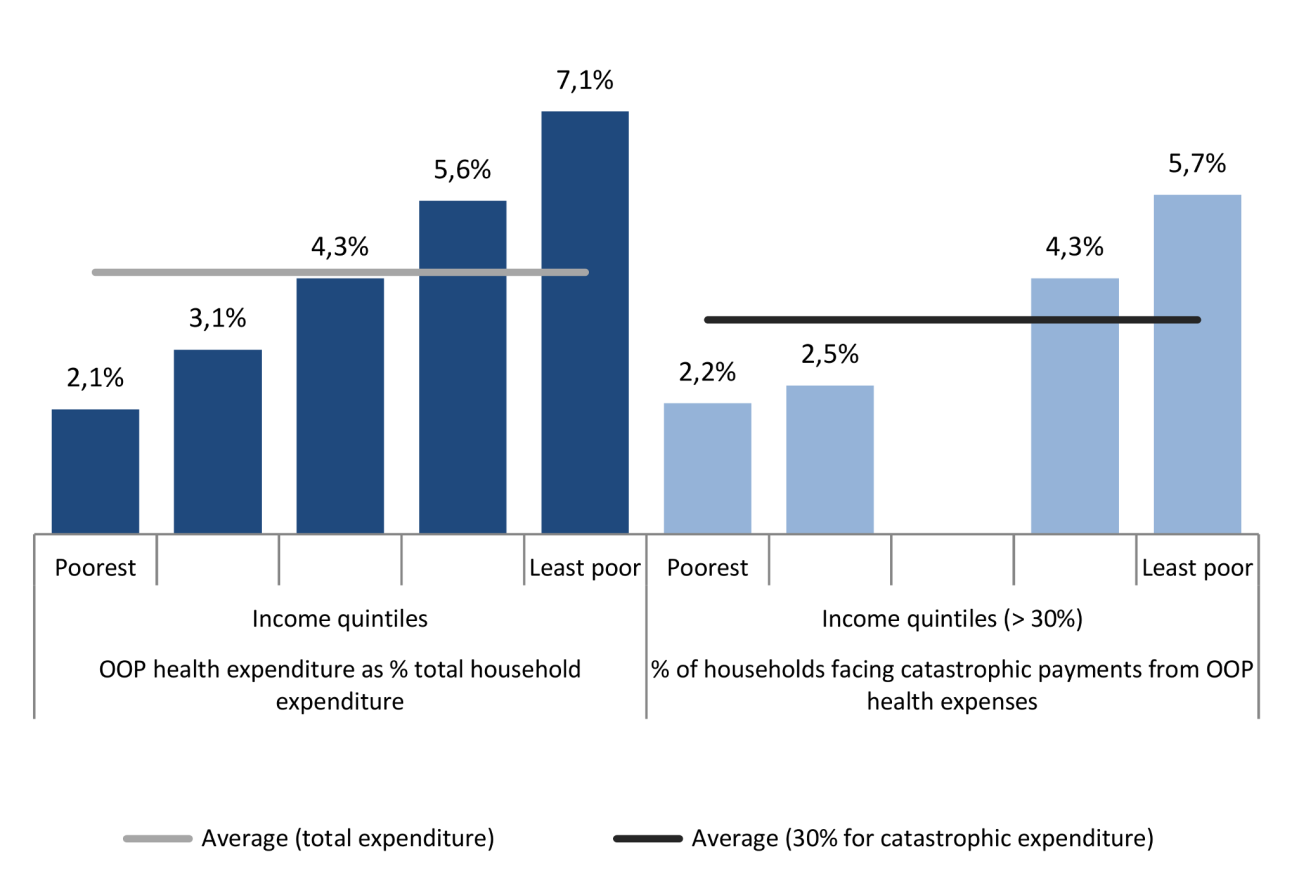


**Figure S2**: Financial Protection Tracer Indicators

Data Source: Authors based on[41]. No data presented for Quintile 3 in the article.

Health Services Result Framework:

In terms of financial resources for health, in 2012, according to OECD, the country spent 7.6% of its GDP on health, being the 44.5% public health expenditure (PHE). The total per capita health expenditure increased from PPP $396 in 1995, to PPP $1,711 in 2012[39]. In the case of physical and human resources, the Chilean situation is far behind the average of the OECD countries (Figure S3).


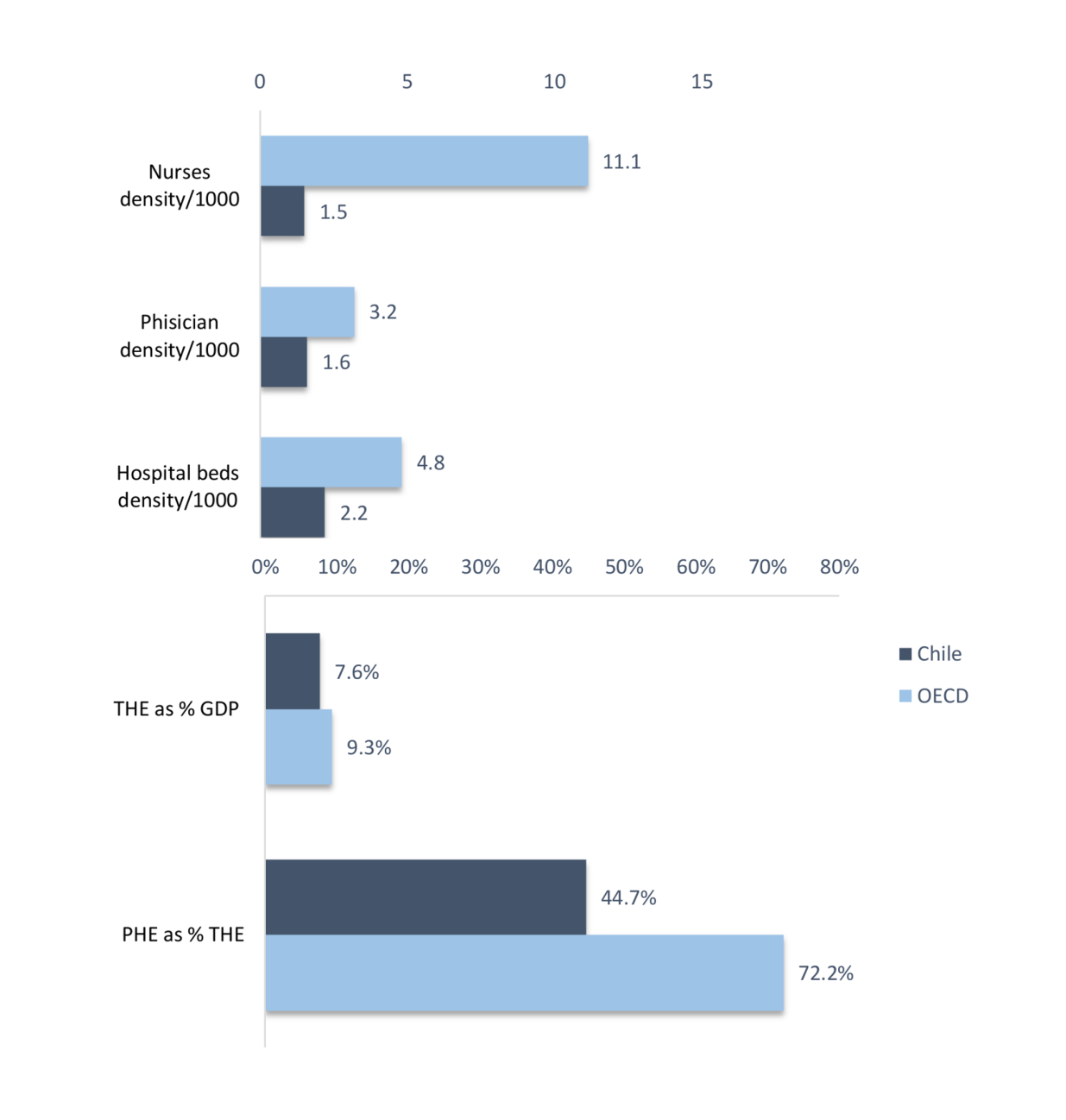


**Figure S3:** Inputs comparison, Chile and OECD 2011

Data Source: authors based on OECD Statistics[39].

Regarding utilization, although in basic services such as doctor consultation the utilization does not show an unequal pattern across income quintile groups, there is an important gradient, favouring the wealthiest, in the case of specialist consultation and laboratory exams; on the contrary, the worse-off show a higher utilization of emergency room visit (Figure S4).


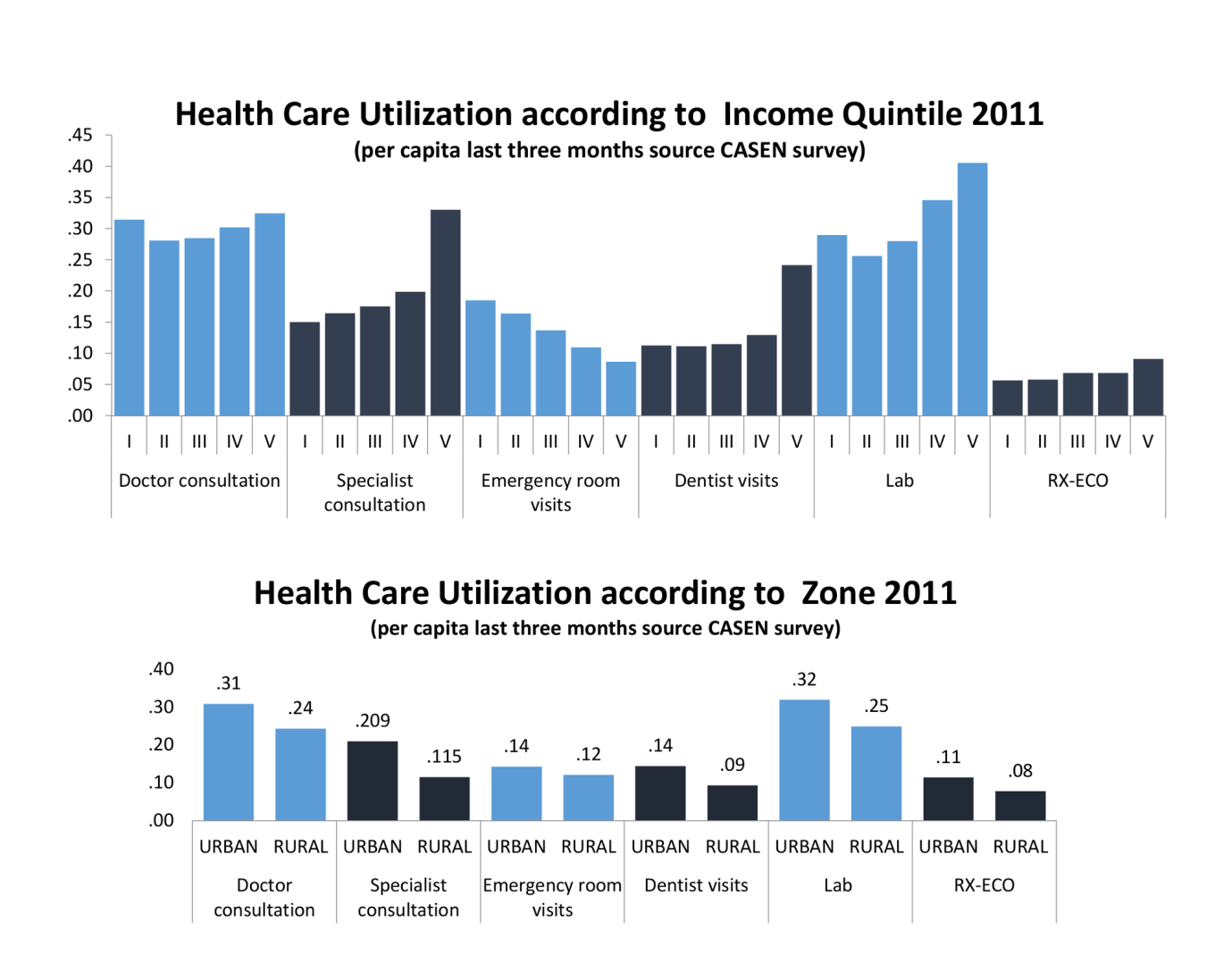


**Figure S4**: Health Care Utilization according to Income

Data Source: Authors based on CASEN 2011.

A similar effect can be seen comparing health care utilization according to residence where residents in rural areas show systematically a lower utilization rate (Figure S5).


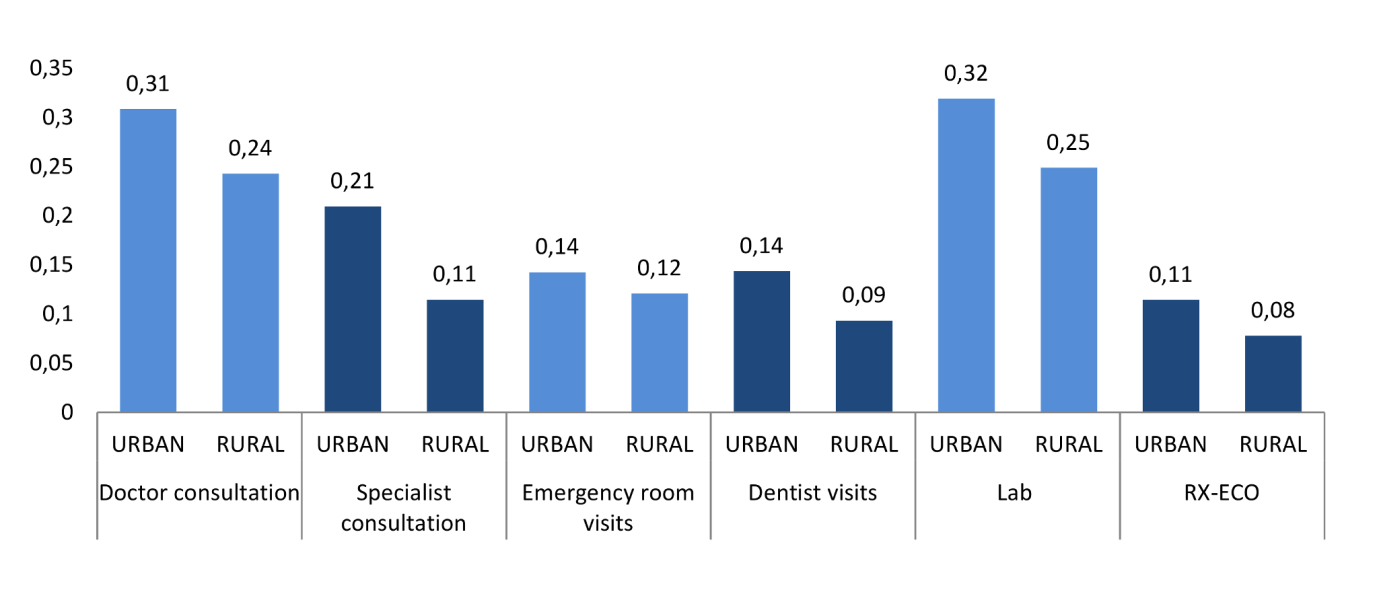


**Figure S5:** Health Care Utilization according to Residence

Data Source: Authors based on CASEN 2011.

In terms of health impact indicators, as it was mentioned before, are characterized by relative good performance in average, but with significant inequalities mainly according to socioeconomic position and residence area[15, 16]. For instance, as shown in Figure S6 significant gradients in infant mortality rate (IMR) can be observed according to women education and residence. The IMR ratio was 2.3 between the least and highest educated women, and 1.4 between rural and urban residence. Similar disparities are observed in risk factors prevalence like overweight, obesity, alcohol use disorders and cardiovascular risks, affecting disproportionally the less educated. In contrast, tobacco user’s prevalence is lower among less educated, rural residents and in people covered by FONASA (Figure S7).

**
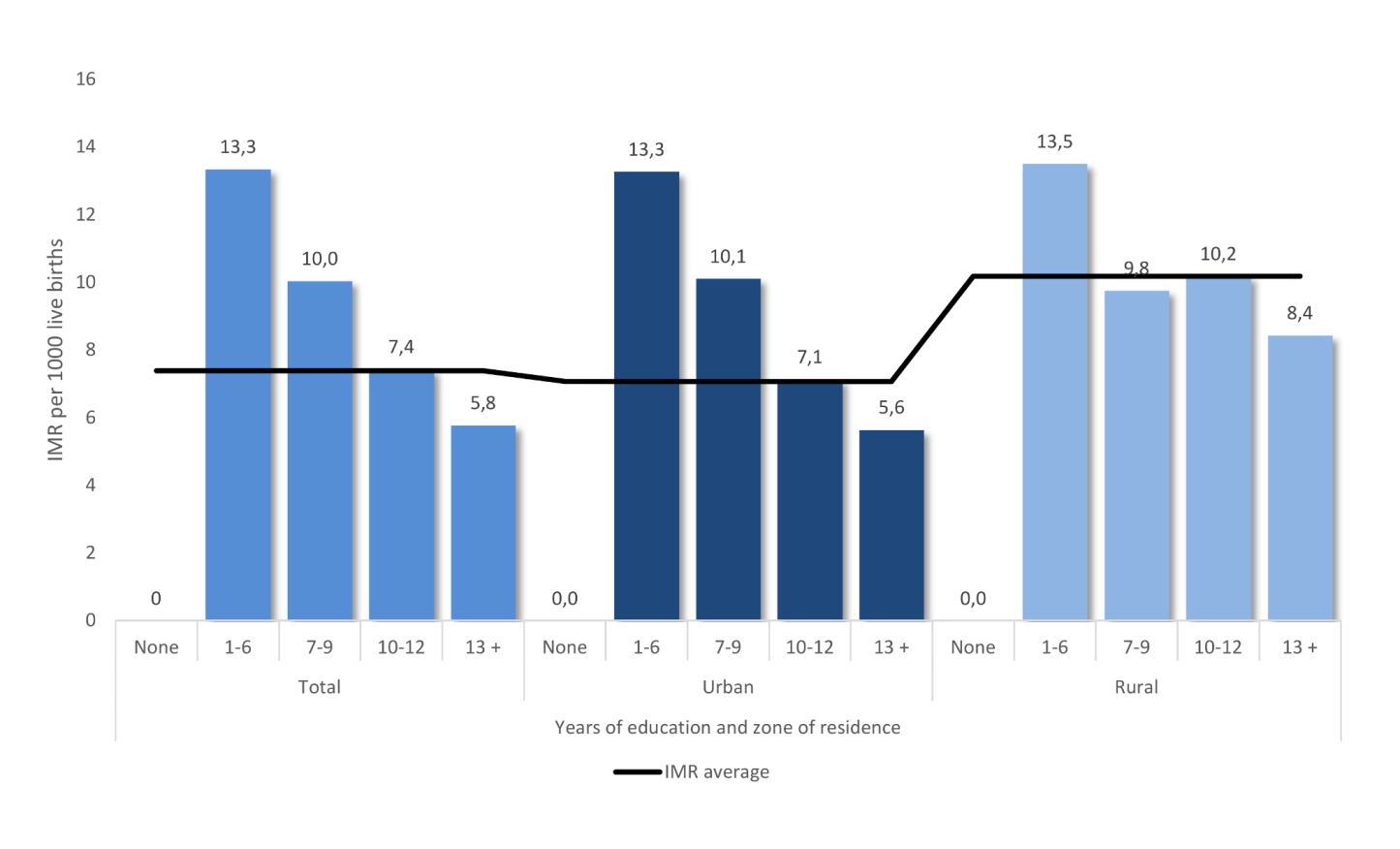
Figure S6:** Infant Mortality Rate according to women education and residence

Data Source: Authors based on Deaths and Live Births Data Base, MOH Chile 2010.


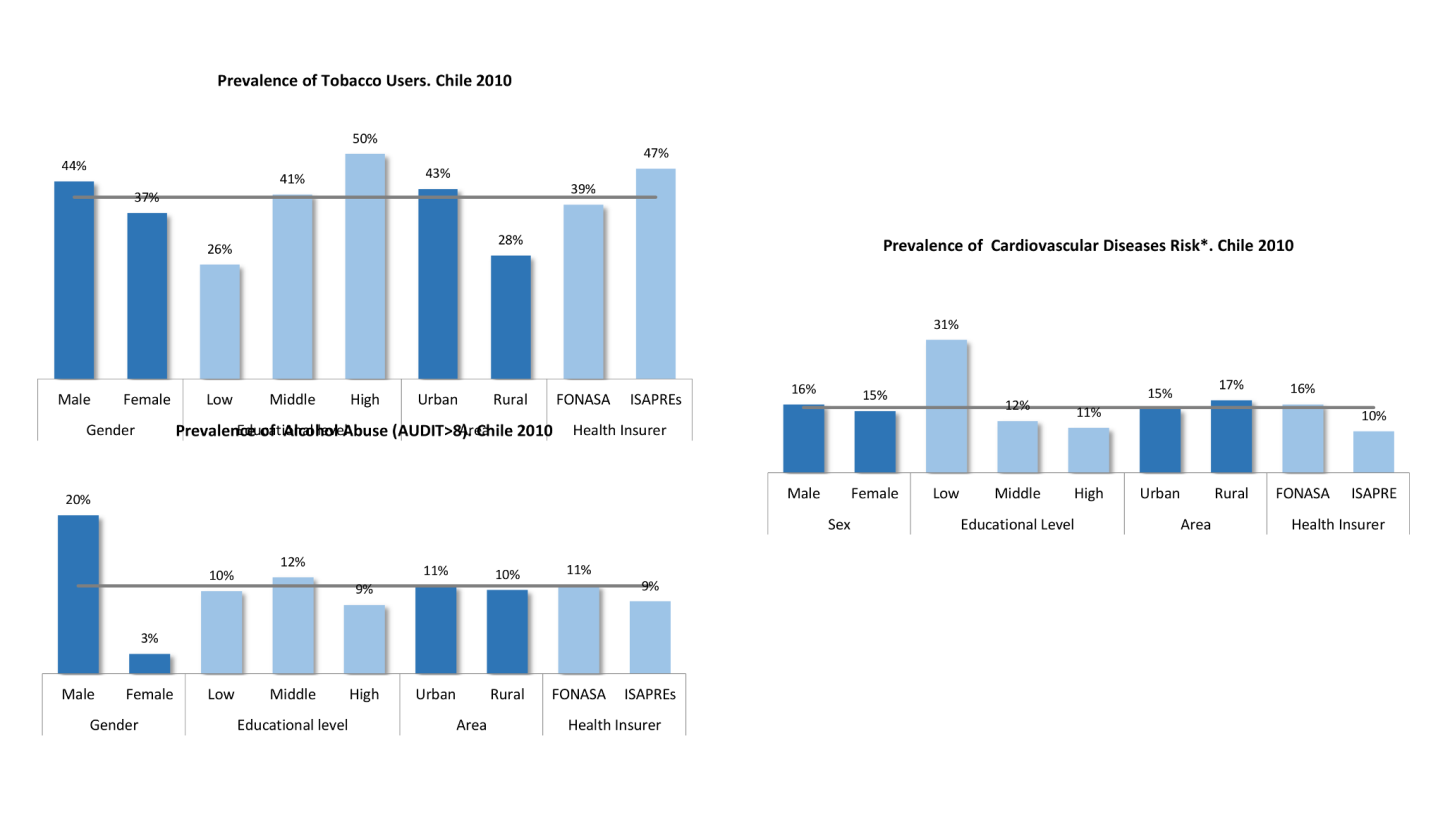


**Figure S7**: Prevalence of Risk Factors. Chile 2010

Data Source: Authors based on 2nd National Health Survey 2009-10. MOH Chile

The demographic and socioeconomic changes have determined the growing importance of non-communicable diseases (NCDs)[27, 28, 42]. In fact, main causes of death are ischemic heart disease, cerebrovascular disease, and cirrhosis of the liver[43]. Between 1990 2010 it was observed a decline in mortality rate at every age range[44].

In 2010 the NCDs accounted for 79% of DALYs in both sexes (86% females; 73% males), showing a 16% of increment in its relative importance since 1990; injuries accounted for 13% (7% females; 18% males), while Group I caused 9% of the burden of diseases - BoD (8% in females vs. 9% in males)[44].

Dietary habits are the leading risk factor, together with high blood pressure, and high body-mass index, all of them are more prevalent in less educated population. Tobacco use continues to be around 40% of the population. The leading risk factors in 2010 for children under 5 and adults aged 15-49 years were suboptimal breastfeeding and alcohol use, respectively. Recently some legislative interventions has been implemented aiming to address risk factors: Raising of tobacco taxes[45]; Restriction to tobacco use[46]; Zero tolerance for alcohol and driving[47]; and the Choose to Live Healthily System (Elige Vivir Sano) to promote healthy habits[48].

**5. Conclusions and recommendations**

It is difficult to state a single judgment about the universality of health coverage in Chile. If UHC were about health insurance coverage and general access to health care facilities, the performance of Chilean system could been judged quite well: 98% coverage of health insurance, and an average of 4% of people that declare not having access to care when they thought they needed it (CASEN 2011). But UHC is attained when needed health services are available and affordable to all the people. This implies a broad spectrum of health services, from prevention to rehabilitation, at a level of quality sufficient to achieve the desired result. It would also require no out-of-pocket payments exceeding the affordability threshold. Having this in mind, another image of the Chilean health system could be seen.

With respect to health services coverage, NCDs are the main causes of BoD in Chile, consequently the interventions addressing these health problems should have more relevance. However coverage is much higher for MDGs diseases and, at the same time risk factors prevalence denotes minimum impact of preventive interventions. Similarly, there are still some gaps in services that address some increasingly important diseases, such as dementias or other with catastrophic cost, like inherited metabolic disorders. Equity monitoring shows lower coverage for males, less educated people, lower income quintiles, resident from rural areas and people ascribed to public health insurance.

Concerning financial protection, the higher risk of facing catastrophic expenditure among richer families could be unexpected, but it can be partly because of the copayment exemption for the worse off and the higher utilization of more complex and expensive services among the better off. In fact equity gaps favouring the wealthiest for more complex interventions, has been reported in previous studies about health care access and utilization in Chile[11, 12, 13, 14].

In summary, over the last 90 years the Chilean health system has moved towards UHC, evidenced by the high coverage on health insurance and relative good performance on health indicators on average. However there are structural constraints that prevent further advance and create gaps and important inequities in terms of services coverage, impacting health results[49]. Moreover, it is observed that the Chilean population is not adequately protected from the risk of facing catastrophic health expenditures.

The application of the health services result framework illustrates the context in which the UHC measurements are obtained. There are important limitations in terms of the inputs or resources available for health, far behind the availability in developed countries and especially in the public sector (human resources and facilities). For instance, the PHE is one of the lowest among OECD countries. Furthermore, an important component of the Total Health expenditure (THE) is financed through OOP expenditure, that correlates with the important proportion of family income devoted to health.

In addition, the system is fragmented, at the pooling and providers level, not allowing for a unique pooling of funds. Therefore, there is an unequal availability of funds for the population served in both health insurance sectors. More to the point, there is risk selection: ISAPREs receives the compulsory payment from their contributors, who represent the richest, healthiest and youngest segment of the population compared to the public insurer (FONASA)[50, 51, 52].

Concerning the measurement and monitoring UHC feasibility, the Chilean health information system provides data for all required aspects. Most data used come from official sources, both from systematic records and population surveys, each one with strengths and limitations.

The health statistic systems regularly report data from systematic records to institutions such as OECD or WHO, ensuring accuracy and comparability. It draws attention data gaps for population in need of priority health interventions, such as antenatal care, and the lack of relevant coverage disaggregation for child vaccination, TB treatment success rate and ART therapy.

Population surveys, conversely, provide disaggregation for equity monitoring. But, there are more concerns about the comparability of this data; discontinuity of surveys, changes in measurement tools and in the representativeness of samples, has affected consistency and periodicity of measures. On the other hand, FP figures only represent the city of Santiago.

UHC monitoring through indicators is feasible but requires robust information systems, periodic health surveys and focus in a vulnerable population, especially in rural areas. Overall, Chile has relevant and useful information that allows measuring and monitoring the path towards UHC.

To increase the resources available for health, to establish solidarity among public and private sectors and to Improve quality of care to expand effective coverage, seems key components towards UHC in Chile (Box S4).

| **Box S4: Recommendations**  To move forward UHC:   - Increase resources available for health, including human, physical infrastructure and financial resources, with an equity focus. - Increase PHE, reducing the share of out of pocket payment. - Establish solidarity among public and private health funds. - Expand financial coverage for medicines, in a context of rational use. - Improve quality of care to expand effective coverage, with a focus on priority services. - Expand services coverage to priority and catastrophic cost diseases.   For UHC measurement and monitoring:   - Investment in research to improve measurement methods, tools and reporting, with a focus on population in need. - Include relevant equity breakdowns in systematic records, including facility reports. - Implement data quality assurance mechanisms. - Ensure the sustainability of the MOH system of routine population health surveys, guaranteeing periodicity and comparability of results. - Coordinate surveys with other governmental institutions that are related to social determinants of health, or contain relevant questions to health sector coverage. |
| --- |

**Acknowledgments:**

The authors would like to thank Gisela Alarcón, Pietro Cifuentes, David Debrott, Consuelo Espinosa and Patricia Frenz, for their advices in the early stages of this review.

**References**

1. WHO. (2000). The World Health Report 2000: health systems - improving performance. Geneva, World Health Organization, 2000.
2. WHO. (2005). Sustainable health financing, universal coverage and social health insurance. World Health Assembly Resolution 58.33, 2005.
3. United Nations. (2012). Global Health and Foreign Policy. UN General Assembly Resolution (A/67/L.36).
4. Vega J. (2013). “UHC: The Post-2015 Development Agenda.” Lancet 381: 179.
5. Mardones-Restar F, Azevedo A. (2006). The essential health reform in Chile; a reflection on the 1952 process. Salud Pública de México, 48(6): 504-511.
6. Berrecil-Montekio V, Reyes JD, Manuel A. (2011). Sistema de Salud de Chile. Salud Pública Mex, 53 supl 2: SI 32- SI 43.
7. FONASA. (2013). Estadísticas FONASA 2009: Demografía. [Available at: http://www.fonasa.cl/wps/wcm/connect/03c92fb1-3e3d-4171-bcb5-15d69d868686/01+- Demograf %C3%ADa_pagina_web_%2822-08- 011%29JAV.xls?MOD=AJPERES&atta chment=true&id=1313788185537l ]. [Accessed on September 23, 2013].
8. Arteaga O, Astorga I, Pinto AM. (2002). Inequalities in Public Health Care Provision in Chile. Cadernos de saúde pública, 18(4): 1053–66. http://www.ncbi.nlm.nih.gov/pubmed/12118310 (November 15, 2013).
9. Frenz P, Delgado I, Kaufman JS, Harper S. (2013). Achieving Effective Universal Health Coverage with Equity: Evidence from Chile. Health policy and planning. http://www.ncbi.nlm.nih.gov/pubmed/23921988 (November 15, 2013).
10. Núñez A, Chi Ch. (2013). Equity in Health Care Utilization in Chile. International journal for equity in health 12(1): 58. http://www.ncbi.nlm.nih.gov/pubmed/23937894 (September 11, 2013).
11. Paraje G, Vásquez F. (2012). Health Equity in an Unequal Country: The Use of Medical Services in Chile. International journal for equity in health 11: 81. http://www.pubmedcentral.nih.gov/articlerender.fcgi?artid=3544610&tool=pmcentrez&rendertype=abstract (November 15, 2013).
12. INE - National Institute of Statistics. (2013). Demographic and Life Statistics. Population Projections. [available at: http://www.ine.cl/canales/chile_estadistico/demografia_y_vitales/proyecciones/MenPrincOK.xls ].
13. World Bank. Statistics. (2013). [available at: http://data.worldbank.org/indicator?display=default].
14. MDS - Ministry of Social Development. (2012a). Poverty Results CASEN 2011. [available at: http://observatorio.ministeriodesarrollosocial.gob.cl/layout/doc/casen/pobreza_casen_2011.pdf].
15. MDS - Ministry of Social Development. (2012b). Education Results CASEN 2011. [available at: http://observatorio.ministeriodesarrollosocial.gob.cl/layout/doc/casen/2013_04_01_Educacion_CASEN_2011.pdf].
16. Ruiz-Tagle J. (1999). Chile: 40 años de desigualdad de ingresos. Departamento de Economía, Universidad de Chile.
17. MDS - Ministry of Social Development. (2012c). Income Distribution Results CASEN 2011. [available at: http://observatorio.ministeriodesarrollosocial.gob.cl/layout/doc/casen/001_ingreso_2011.pdf].
18. Chilean Government. (2011). National Health Strategy 2011-2020 (Estrategia Nacional de Salud para el Cumplimiento de los Objetivos Sanitarios de la Década 2011-2020).
19. MoH – Ministry of Health. 2010. Evaluación de Los Objetivos Sanitarios para la Década 2000-2010. 1st ed. ed. Claudia Gonzalez. Ministerio de Salud de Chile.
20. Molina H, Cordero M, Silva V. (2008). De la sobrevida al desarrollo integral de la infancia: Pasos en el desarrollo del sistema de protección integral a la infancia. Rev Chil Pediatr; 79 Supl (1): 11-17.
21. Chilean Government. (2013). Elige Vivir Sano (Choose to Live Healthily). [Internet; available at: http://www.gob.cl/english/elige-vivir-sano-choose-to-live-healthily-campaign/] [Accessed on August 17, 2013].
22. Library of Congress. (2004). History of Law N°19.966. Establishes the Explicit Health Guarantees Regime. Ministry of Health. Chilean Government. Document in Spanish.
23. Superintendencia de Salud. (2011). Análisis de los Planes de Salud del Sistema ISAPRE. Enero 2011. Departamento de Estudios y Desarrollo.
24. MoH – Ministry of Health. (2010). Primera Encuesta Nacional de Empleo, Trabajo y Salud. ENETS 2009. [available at: http://epi.minsal.cl/wp-content/uploads/2012/07/Resumen_Ejecutivo_ENETS_Final.pdf].
25. MoH – Ministry of Health. (2006). II Quality of Life and Health Survey. Final report. Ministry of Health, Chile. Document in Spanish. [available at: http://epi.minsal.cl/wp-content/uploads/2012/07/Informe-Final-Encuesta-de-Calidad-de-Vida-y-Salud-2006.pdf].
26. MoH – Ministry of Health. (2000). I Quality of Life and Health Survey. Report on Family Planning. [available at: http://epi.minsal.cl/wp-content/uploads/2012/07/infplanfam.pdf].
27. MoH – Ministry of Health. (2010). Results of the National Health Survey 2009-2010. Final Report. Document in Spanish. Ministry of Health, Chile. Document in Spanish. [available at: http://www.encuestasalud.cl/].
28. MoH – Ministry of Health. (2003). Results of the National Health Survey 2003. Final Report. Document in Spanish. Ministry of Health, Chile. Document in Spanish [available at: http://epi.minsal.cl/epi/html/invest/ENS/InformeFinalENS.pdf].
29. MoH – Ministry of Health. (2006). Satisfaction and Out-of-Pocket Payment Survey. Final Report on Satisfaction with the Health System. Ministry of Health, Chile. Document in Spanish. [available at: http://epi.minsal.cl/epi/0notransmisibles/gasto-salud/Informe_final_Satisfaccion.pdf].
30. MoH – Ministry of Health. (2006). Satisfaction and Out-of-Pocket Payment Survey. Final report on Out of Pocket Expenditure. Ministry of Health, Chile. Document in Spanish. [available at: http://desal.minsal.cl/PUBLICACIONES-2007.html].
31. National Youth Institute (INJUV). (2012). Séptima Encuesta Nacional de Juventud. [available at: http://www.injuv.gob.cl/portal/wp-content/files_mf/septimaencuestanacionaljuventud2.pdf ].
32. WHO (2010). Health systems financing: the path to universal coverage. Geneva, World Health Organization.
33. Evans D, Saksena P, Elovainio R, Boerma T. (2012). Measuring Progress towards Universal Coverage. July 2012. WHO, Geneva.
34. The World Bank. (2008). Governing a hybrid mandatory health insurance system: The case of Chile. Bitrán R, Muñoz R, Escobar L, Farah C. Chapter 6 in Governing Mandatory Health Insurance: Learning from Experience. Edited by Savedoff W, Gottret P.
35. MoH – Ministry of Health. (2008). Verification of the expected cost by beneficiary of the health problem with explicit guarantees: Birth Attendance. Final Report. Prepared by Bitran & Asociados. [available at: http://desal.minsal.cl/wp-content/uploads/2013/09/Informe-Final-Partos-GES-9-abril-2008.pdf].
36. Herrera T. (2013). La Situación de La Tuberculosis En Chile y Los Actuales Desafíos. Visita de La OPS Al Programa de Control de La Tuberculosis de Chile. Rev Chil Enf Respir, 29: 46–49.
37. Tanahashi T. (1978). Health Service Coverage and Its Evaluation. Bull World Health organization 56(2): 295–303.
38. Intendencia de Fondos y Seguros Previsionales. (2012). Informe de Fiscalización Examen de Medicina Preventiva Julio 2011 – Junio 2012. http://www.supersalud.gob.cl/portal/articles-8426_recurso_1.pdf.
39. OECD Statistics. (2013). OECD Health Data 2013. Frequently Requested Data. [available at: http://www.oecd.org/health/health-systems/oecdhealthdata2013-frequentlyrequesteddata.htm].
40. Castillo-Laborde C, Villalobos Dintrans P. (2013). Caracterización del gasto de bolsillo en salud en Chile: Una mirada a dos sistemas de protección. Revista Médica de Chile, 141: 1456-1463.
41. Cid C, Prieto L. (2012). El gasto de bolsillo en salud: el caso de Chile, 1997 y 2007. Revista Panamericana de Salud Pública, 31(4): 310-316.
42. Villalón G, Vera S. (2012). Panorama demográfico en Chile contemporáneo: desafíos para la sociedad del siglo XXI. Anales de la Universidad de Chile, Séptima Serie N°3.
43. MoH – Ministry of Health. (2013). Estadísticas de Mortalidad. [ available at: http://www.deis.cl/wp-content/uploads/2012/10/10-primeras-causas-de-muerte-Chile-2000-2010.xlsx]
44. IHME - Institute of Health Metrics. (2013). GBD Profile: Chile. Global Burden of Diseases, Injuries, and Risk Factors Study 2010. [available at: http://www.healthmetricsandevaluation.org/sites/default/files/country-profiles/GBD Country Report - Chile.pdf] . [Accessed on August 30, 2013].
45. Library of Congress. (2010). Law N°20.455. Amending various legal bodies intended to obtain resources for the country reconstruction funding. Ministry of Finance. Chilean Government. Document in Spanish.
46. Library of Congress. (2013). Law N°20.660. Amends Law N°19.419, in matters of free smoke environments. Ministry of Health. Chilean Government. Document in Spanish.
47. Library of Congress. (2012). Law N°20.580. Amends Law N°18.290, increasing the penalties for driving while drunk, under the influence of narcotic or psychotropic substances, and under the influence of alcohol. Ministry of Transport and Telecommunications. Chilean Government. Document in Spanish.
48. Library of Congress. (2013). Law N°20.670. Creates the Choose to Live Healthily System. Ministry of Social Development. Chilean Government. Document in Spanish.
49. Hollstein RD, Vega J, Carvajal J. (1998). Social Inequalities and Health. Socioeconomic Level and Infant Mortality in Chile in 1985-1995. Revista Médica de Chile, 126(3): 333–40. http://www.ncbi.nlm.nih.gov/pubmed/9674305 (November 15, 2013).
50. Pardo C, Schott W. (2013). Health Insurance Selection in Chile: a Cross-Sectional and Panel Analysis. Health policy and planning: czt017–. http://heapol.oxfordjournals.org/content/early/2013/04/03/heapol.czt017.long (November 15, 2013).
51. Bastías G, Pantoja T, Leisewitz T, Zarate V. (2008). Health care reform in Chile. CMAJ, 179(12): 1289-1292.
52. Kutzin J. (2001). A descriptive framework for country-level analysis of health care financing arrangements. Health Policy, 56(3): 171-203.
53. MoH – Ministry of Health. (2013). Decreto N°4. Aprueba Garantías Explícitas en Salud del Régimen General de Garantías en Salud. [available at: http://www.minsal.cl/portal/url/item/d6924d33612dd5e6e040010164015e8f.pdf].
54. Bitrán R. (2013). Explicit Health Guarantees for Chileans: The AUGE Benefits Package. UNICO Studies Series 21, Washington DC, January 2013.

| **Box S5: Health problems covered by the GES** ^[53, 54]^  1. Chronic renal failure stages 3 and 4  2. Operable congenital heart disease (under 15 years of age)  3. Cervical cancer  4. Pain relief and palliative care for advanced cancer  5. Acute Myocardial Infarction  6. Diabetes Mellitus Type I  7. Diabetes Mellitus Type II  8. Breast cancer (15 years of age or more)  9. Spinal Dysraphia  10. Surgical treatment of scoliosis (under 25 years of age)  11. Cataract surgery  12. Total hip replacement in people with severe hip osteoarthritis (65 years of age or more)  13. Cleft lip and palate  14. Cancer in people under 15 years of age  15. Schizophrenia  16. Testicular cancer (15 years of age or more)  17. Lymphoma (15 years of age or more)  18. HIV/AIDS  19. Ambulatory care of Acute Respiratory Infection - ARI (under 5 years of  age)  20. Ambulatory care of community-acquired pneumonia (65 years of age or  more)  21. Primary or essential arterial hypertension  22. Non-refractory epilepsy (1 to 15 years of age)  23. Comprehensive oral health (6  years old)  24. Prevention of pre-term delivery  25. Disorders of impulse generation and conduction in persons requiring pacemaker (15 years of age or  more)  26. Gallbladder cancer preventive cholecystectomy  27. Gastric cancer  28. Prostate cancer  29. Refraction errors (65 years of age or more)  30. Strabismus (under 9 years of age)  31. Diabetic retinopathy  32. Rhegmatogenous and non-traumatic retinal detachment  33. Hemophilia  34. Depression (15 years of age or more)  35. Treatment of benign prostatic hyperplasia in symptomatic persons  36. Orthesis (or technical aids) (65 years of age or more)  37. Ischemic stroke (15 years of age or more)  38. Ambulatory care of chronic obstructive pulmonary disease – COPD  39. Moderate and severe asthma  40. Newborn respiratory distress syndrome  41. Medical treatment of mild and moderate osteoarthritis of hip and/or knee (55 years of age or more)  42. Subarachnoid bleeding secondary to ruptured cerebral aneurysms  43. Primary tumors of the central nervous system  44. Surgical treatment of lumbar herniated nucleus pulposus  45. Leukemia (15 years of age and more)  46. Outpatient dental emergency  47. Comprehensive oral health (60  years old)  48. Severe multiple trauma  49. Moderate or severe traumatic brain injury  50. Severe eye trauma  51. Cystic fibrosis – CF  52. Rheumatoid arthritis  53. Harmful consumption or dependency on alcohol and drugs (low to moderate risk) (under 20 years of age)  54. Labor analgesia  55. Major burn patients  56. Bilateral hearing loss in people who need hearing aids  57. Retinopathy of prematurity  58. Bronchopulmonary dysplasia of prematurity  59. Bilateral sensorineural hearing loss of prematurity  60. Non-refractory epilepsy (15 years of age or more)  61. Bronchial asthma (15 years of age or more)  62. Parkinson disease  63. Juvenile idiopathic arthritis  64. Secondary prevention of terminal chronic renal failure  65. Hip dysplasia  66. Comprehensive oral health of the pregnant woman  67. Relapsing remitting multiple sclerosis  68. Chronic hepatitis B  69. Hepatitis C  70. Colorectal cancer (15 years of age and more)  71. Epithelial ovarian cancer  72. Gallbladder cancer (15 years of age and more)  73. Osteosarcoma (15 years of age and more)  74. Surgical treatment of chronic lesions of aortic valve  75. Bipolar disorder (15 years of age and more)  76. Hypothyroidism (15 years of age and more)  77. Treatment of moderate hearing loss (under 2 years of age)  78. Systemic lupus erythematous  79. Surgical treatment of chronic lesions of mitral and tricuspid valve  80. Treatment for Helicobacter pillory eradication |
| --- |

| **Intervention area** | **Tracer indicators of intervention coverage** | **Average** | **Equity dissagregation** | | | | | | | | | | | | | | | **Source** |
| --- | --- | --- | --- | --- | --- | --- | --- | --- | --- | --- | --- | --- | --- | --- | --- | --- | --- | --- |
|  |  |  | **Health insurance** | | | **Household income** | | | **Educational level** | | | **Residence** | | | **Gender** | | |  |
|  |  |  | **Public FONASA** | **Private ISAPRE** | **ratio** | **Q1** | **Q5** | **ratio** | **Low: <8 YED** | **High: >12 YED** | **ratio** | **Rural** | **Urban** | **ratio** | **Male** | **Female** | **ratio** |  |
| **Health-related MDGs or Group I BoD** | |  |  |  |  |  |  |  |  |  |  |  |  |  |  |  |  |  |
| Pregnancy care | Antenatal care (at least one visit).The percentage of women with a live birth that received antenatal care provided by skilled health personnel at least once during their pregnancy. | 96% | 97% | 91% | 1.1 | n/a | n/a | - | n/a | n/a | - | n/a | n/a | - | - | - | - | [35] Estudio de Verificación de Costos: Atención del Parto. MOH Chile 2008 |
| Maternal and newborn care | Skilled birth attendance, % of pregnant women | 100% | n/a | n/a | - | n/a | n/a | - | n/a | n/a | - | n/a | n/a | - | - | - | - | MOH Chile, 2010 |
| Family planning | Proportion of FP need satisfied | 99% | 99% | 100% | 1 | n/a | n/a | - | 99% | 100% | 1 | 98% | 99% | 1 | 99% | 100% | 1 | Quality of Life and Health Survey (ENCAVI 2000). MOH Chile. |
| Child vaccination | Pentavalent3 coverage (diphtheria, tetanus, whooping cough, hepatitis B and Haemophilus influenza type b): % of population immunized (less than 1 year old) | 94% | n/a | n/a | - | n/a | n/a | - | n/a | n/a | - | 93% | 100% | 0.9 | n/a | n/a | - | Departament of statistics "Cobertura de inmunizaciones 2005-10". MOH-Chile |
| TB control | Treatment success rate, (cure rate among estimated cases) | 82% | n/a | n/a | - | n/a | n/a | - | n/a | n/a | - | 74% | 91% | 0.8 | n/a | n/a | - | National TB Program MOH-Chile |
| HIV prevention & treatment | Use of barriers methods during last sexual intercourse. Proportion of population 15-29 years [%] | 49% | n/a | n/a | - | 45% | 58% | 0.8 | n/a | n/a | - | n/a | n/a | - | 59% | 37% | 1.6 | INJUV Chile. 7th National Youth Survey 2012* |
|  | PMTCT among HIV positive women | 89% | 94% | n/a | - | n/a | n/a | - | n/a | n/a | - | n/a | n/a | - | n/a | n/a | - | national Program HIV-AIDS-STD. MOH Chile, 2011 |
|  | ART coverage | 64% | n/a | n/a | - | n/a | n/a | - | n/a | n/a | - | n/a | n/a | - | n/a | n/a | - | national Program HIV-AIDS-STD. MOH Chile, 2012 |
| Water and sanitation | Adequate sanitation: Proportion of household using improved drinking water sources and improved sanitation facilities [%] | 92% | 91% | 98% | 0.9 | 86% | 97% | 0.9 | 86% | 98% | 0.9 | 62% | 96% | 0.6 | 91% | 93% | 1 | CASEN Survey 2011. Ministry of Social development Chile |
| **Chronic conditions & injuries (CCI) or Group II and III BoD** | |  |  |  |  |  |  |  |  |  |  |  |  |  |  |  |  |  |
| Chronic adult conditions | Effective coverage of High Blood Pressure: Normal BP among those in need of hypertension treatment [%] | 17% | 18% | 13% | 1.4 | n/a | n/a | n/a | 16% | 17% | 1 | 17% | 17% | 1 | 9% | 25% | 0.4 | 2nd National Health Survey 2009-10. MOH Chile |
|  | Effective coverage of Diabetes : Normal glycemia among those in need of diabetes treatment [%] | 35% | 34% | 42% | 0.8 | n/a | n/a | n/a | 35% | 38% | 0.92 | 23% | 37% | 1.6 | 29% | 39% | 0.7 | 2nd National Health Survey 2009-10. MOH Chile |
|  | Depression treatment: people under treatment among those with depressive symptoms during the last year the last - ICID (>15 years) [%] | 58% | 56% | 82% | 0.7 | n/a | n/a | n/a | 60% | 67% | 0.9 | 53% | 58% | 1.1 | 35.90% | 64.60% | 0.6 | 2nd National Health Survey 2009-10. MOH Chile |
| Cancer | Cervical cancer screening: proportion of women (15 years and older) with PAP smear during last 3 years [%] | 56% | 55% | 61% | 0.9 | 55% | 61% | 0.9 | 51% | 59% | 0.88 | 57% | 55% | 1 | - | - | - | CASEN Survey 2011. Ministry of Social development Chile |
|  | Mammography: Proportion of women (50 years and older) with mammography in the last 3 years [%] | 54.40% | 52.20% | 77.20% | 0.7 | 43.70% | 70.20% | 0.6 | 43.60% | 66.90% | 0.65 | 48.30% | 55.20% | 1.1 | - | - | - | CASEN Survey 2011. Ministry of Social development Chile |
| Injuries | Trauma care within specific time span | n/a |  |  |  |  |  |  |  |  |  |  |  |  |  |  |  |  |
| Notes: |  |  |  |  |  |  |  |  |  |  |  |  |  |  |  |  |  |  |
| n/a= not available | | | | | | | | | | | | | | | | | | |
| YED = years of education | | | | | | | | | | | | | | | | | | |
| Underlined figures: |  |  |  |  |  |  |  |  |  |  | | | | | |  |  |  |
| Antenatal care: Disagregation according to public and private sector is considering the provider level instead of health insurance | | | | | | | | | | | | | | | | | | |
| Child vaccination: A proxy for rural and urban population was estimated. Rural= average of the country´s administrative regions with >25% of rural population. Urban= average of regions with <25% of rural population. | | | | | | | | | | | | | | | | | | |
| TB treatment success: urban and rural coverage is not available, the data reflect the administrative region with the highest and lower TB treatment success | | | | | | | | | | | | | | | | | | |
| HIV prevention & treatment: ART coverage following recommendation of TARV for CD4 of 500 cells/mm3 or less | | | | | | | | | | | | | | | | | | |
| *7th National Youth Survey use Socioeconomic Status instead of Household Income Quintiles. The data reported in the table corresponds to Low SES and High SES | | | | | | | | | | | | | | | | | | |

**Table S2:** Intervention Coverage Indicators

Data Source: Authors, based on several sources
